# Supplementary material for: Improving Reporter Gene Assay Methodology for Evaluating the Ability of Compounds to Restore P53 Activity
Source: Int J Mol Sci. 2022 Nov 10;23(22):13867. doi: 10.3390/ijms232213867 (PMC9694221; doi:10.3390/ijms232213867)
Supplement: Supplementary file 1 [file ijms-23-13867-s001.zip › ijms-1876586-supplementary.pdf]

Supplementary Materials

Supplementary Figure

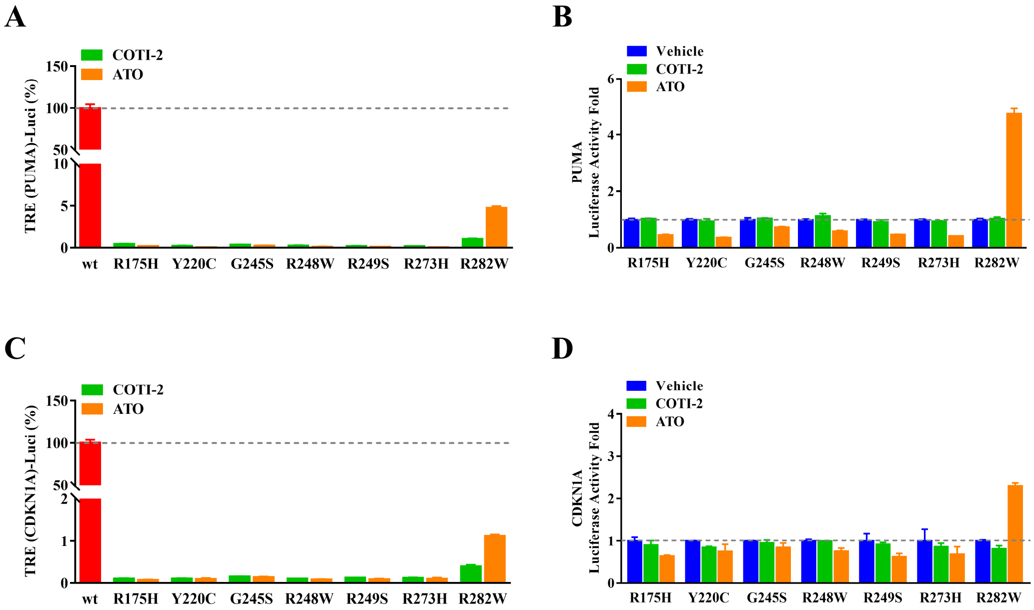

Figure S1. Comparison between two computational logics for reporter gene data. Activity percentage calculated with wtP53 as 100% activity reference (A and C) and fold change calculated with solvent control as the denominator (B and D) by using PUMA and CDKN1A reporter gene, respectively.

Supplementary Table

Table S1. Primers for Real-time qPCR

Primer	Sequence (5'to 3')
TP53-F	TTTCACCCTTCAGATCCGTGG
TP53-R	TATGGCGGGAGGTAGACTGA
PUMA-F	GACGACCTCAACGCACAGTA
PUMA-R	GGTAAGGGCAGGAGTCCCAT
CDKN1A-F	TTAGCAGCGGAACAAGGAGT
CDKN1A-R	CGTTAGTGCCAGGAAAGACAAC
MDM2-F	ATCAGGCAGGGGAGAGTGAT
MDM2-R	CAATTCTCACGAAGGGCCCA
BAX-F	GATGCGTCCACCAAGAAGCT
BAX-R	CGGCCCCAGTTGAAGTTG
β-actin-F	GAGCACAGAGCCTCGCCTTT
β-actin-R	TCATCATCCATGGTGAGCTGGC

Table S2. P53 target gene and the binding site sequences.

P53 target gene	DNA sequence (5'to 3')
CDKN1A	GAACATGTCCCAACATGTTG
PUMA	CTGCAAGTCCTGACTTGTCC
MDM2	GGTCAAGTTGGGACACGTTC
BAX	TCACAAGTTAGAGACAAGCCT
